# miRNA-218 Targets Lipin-1 and Glucose Transporter Type 4 Genes in 3T3-L1 Cells Treated With Lopinavir/Ritonavir

**DOI:** 10.3389/fphar.2019.00461

**Published:** 2019-04-30

**Authors:** Elena Bresciani, Cecilia Saletti, Nicola Squillace, Laura Rizzi, Laura Molteni, Ramona Meanti, Robert J. Omeljaniuk, Giuseppe Biagini, Andrea Gori, Vittorio Locatelli, Antonio Torsello

**Affiliations:** ^1^School of Medicine and Surgery, University of Milano-Bicocca, Monza, Italy; ^2^Division of Infectious Diseases, Department of Internal Medicine, San Gerardo Hospital, Monza, Italy; ^3^Department of Biology, Lakehead University, Thunder Bay, ON, Canada; ^4^Department of Biomedical, Metabolic and Neural Sciences, University of Modena and Reggio Emilia, Modena, Italy; ^5^Infectious Diseases Unit, Department of Internal Medicine, Fondazione IRCCS Ca’ Granda, Ospedale Maggiore Policlinico, University of Milan, Milan, Italy

**Keywords:** HIV protease inhibitors, lipodystrophy, miRNA, adipocyte, insulin resistance

## Abstract

**Background:** Metabolic complications represent a common and serious problem associated with HIV infection and combined Antiretroviral Therapy (cART). Alterations in body fat distribution are associated with significantly increased risks of (i) metabolic derangements, (ii) cardiovascular pathologies, and (iii) insulin resistance. A case control study showed that in subcutaneous adipose tissue from HIV-infected patients on cART presenting lipodystrophy (LS), the levels of miRNA-218 were upregulated and those of lipin-1, a putative target gene of miRNA-218, were downregulated compared with HIV-negative subjects. Lipin-1 is one of the most important factors linked to development of LS. Lipin-1, by controlling PPARγ2, regulates the expression of specific genes, such as that of glucose transporter type 4 (GLUT-4), required for maturation and maintenance of adipocytes.

**Objectives:** To determine whether lopinavir/ritonavir (LPV/RTV) can modulate lipogenesis in adipocytes affecting miRNA-218 and lipin-1 mRNA expression, and to investigate the functional link between miRNA-218 and GLUT-4 mRNA expression.

**Methods:** Differentiated 3T3-L1 cells were treated with various combinations of LPV/RTV, followed by measurements of cell viability, lipid accumulation, lipin-1 and GLUT-4 mRNA and miRNA-218 levels. Transfection of anti-miR-218 or a miRNA-218 mimic were used to investigate the role of miRNA-218 in lipogenesis.

**Results:** LPV/RTV treatment of 3T3-L1 cells did not affect the viability of differentiated 3T3-L1 cells, but caused (i) a significant decrease of lipid accumulation, (ii) an overexpression of miRNA-218, and (iii) a reduction of lipin-1 and GLUT-4 mRNA levels. The anti-miR-218 transfection of 3T3-L1 cells significantly ameliorated the adipogenic dysfunction and restored mRNA levels of lipin-1 and GLUT-4 consequent to LPV/RTV treatment. By contrast, 3T3-L1 cells transfected with a specific miRNA-218 mimic showed (i) an overexpression of miRNA-218, (ii) a reduced cellular lipid fraction, and (iii) decreased levels of mRNA for lipin-1 and GLUT-4.

**Conclusion:** 3T3-L1 cells, treated with LPV/RTV, show altered lipid content due to increased miRNA-218 levels, which affects lipin-1 mRNA. Moreover, increased miRNA-218 levels were inversely correlated with changes in GLUT-4 expression, which suggests a role for miRNA-218 in mediating the insulin resistance consequent to cART.

## Introduction

Metabolic syndrome is a serious consequence of combined Antiretroviral Therapy (cART). HIV-associated metabolic syndrome is often accompanied by lipodystrophy (LS), the redistribution of body fat with loss of subcutaneous adipose tissue in face, limbs and buttocks, concomitant with fat accumulation in the trunk and in the intra-abdominal and inter-scapular regions. These morphological alterations are associated with metabolic dysfunctions including (i) insulin-resistance, (ii) hypercholesterolemia, and (iii) hypertriglyceridemia (Reinsch et al., 2012), all of which predispose the individual to increased risks of developing cardiovascular diseases as well as type II diabetes (Costa and Almeida, 2015).

The causes of HIV-associated metabolic syndrome and LS are multiple, and poorly understood. HIV itself promotes LS, by generating a local inflammation of adipose tissue (Caron-Debarle et al., 2010). Similarly, the nucleoside reverse transcriptase inhibitors (NRTIs) and protease inhibitors (PIs) (Minami et al., 2011; Manente et al., 2012) used in cART, primarily contribute to the development of metabolic dysfunctions by promoting lipohypertrophy and lipoatrophy, respectively, ultimately manifested in an imbalance of adipose tissue functions (Gibellini et al., 2012). Based on the most current guidelines of United States Department of Health and Human Services for Use of Antiretroviral Agents in HIV-infected patients, PIs are still a key component of ART regimens, due the ability to maintain viral load suppression, and will continue to be important drugs for the foreseeable future. In this context, lopinavir (LPV) co-formulated with ritonavir (RTV) in a ratio of 4:1, respectively, represent a drug association frequently associated with metabolic syndrome, that could predispose to fat redistribution, and development of cardiovascular events (Croxtall and Perry, 2010).

Recently, it has been reported that dysregulation of microRNA (miRNA) biogenesis may play a role in the onset of HIV-associated metabolic syndrome and LS (Xu and Sun, 2015). MiRNAs are small non-coding RNAs of about 20-nucleotides in length, regulating gene expression at the posttranscriptional level by binding to the target mRNA, leading either to its degradation or to translational repression (Bartel, 2004). Several miRNAs, i.e., miR-27, miR-130, miR-146b, and miR195a, are involved in the regulation of adipogenesis with a promoting or suppressing role (Lin et al., 2009; Lee et al., 2011; Ahn et al., 2013; Yun et al., 2015), important for maintaining homeostasis of adipose tissue. In subcutaneous adipocytes of HIV-infected patients treated with cART, some miRNAs, including miRNA-218, are upregulated (Squillace et al., 2014). Lipin-1 is among miRNA-218 putative target genes (Squillace et al., 2014), as suggested by bioinformatics analysis and publicly available algorithms that are commonly used to predict downstream targets in microRNA database (Squillace et al., 2014; Han et al., 2015). Lipin-1 encodes a protein with a double function: it is a phosphatidate phosphatase, involved in triglycerides synthesis, and a transcriptional coactivator (Chen et al., 2015) necessary for adipogenesis and the appropriate function of mature adipocytes (Koh et al., 2008). A null mutation in the lipin-1 gene leads to lipodystrophy in mice, and is characterized by (i) loss of adipose tissue, (ii) deficiency in adipocyte differentiation, and (iii) eventually to insulin resistance and circulating hyperlipidemia (Peterfy et al., 2001). Expression of lipin-1 is also inversely correlated with insulin sensitivity: indeed, reduced levels of lipin-1 in mature adipocytes are associated with reduced expression of glucose transporter 4 (GLUT-4) (van Harmelen et al., 2007). Lipin-1 activity is mediated by a direct interaction with PPARγ2, which in turn controls GLUT-4 expression (van Harmelen et al., 2007). The inverse correlation between lipin-1 mRNA levels and miRNA-218 expression in subcutaneous fat samples of HIV-positive subjects suggests a negative regulation on lipin-1 expression by miRNA-218 (Squillace et al., 2014).

In this study we used a simplified model of *in vitro* lipodystrophy, consisting of differentiated 3T3-L1 cells treated with a LPV/RTV co-formulation, in order to investigate the relationship between miRNA-218 and the target gene predicted by bioinformatics analysis. In this model, characterized by decreased levels of lipin-1 mRNA expression and increased levels of miRNA-218, we demonstrate by, respectively, loss and gain of function experiments that miRNA-218 targets the lipin-1 gene, and negatively regulates the mRNA levels of GLUT-4.

## Materials and Methods

### Cell Culture

The murine 3T3-L1 cell line was obtained from the American Type Culture Collection (Manassas, VA, United States) and grown in Dulbecco’s modified Eagle’s medium (DMEM) supplemented with 10% newborn calf serum (NCS) (Euroclone, Pero, Italy), 100 IU/ml penicillin, and 100 μg/ml streptomycin (Invitrogen, Carlsbad, CA, United States). Cells were maintained at 37°C in 5% CO_2_. Sub-confluent cells were induced to differentiate (day 0) by incubation with Induction Medium (DMEM supplemented with 10% fetal bovine serum (FBS, EuroClone, Pero, Italy), 0.5 mM 3-isobutyl-1-methylxanthine, 1 μM dexamethasone and 10 μg/ml insulin) for 2 days. The culture medium was then replaced every 48 h with Insulin Medium (DMEM supplemented with 10% FBS and 10 μg/ml insulin) until the adipocytes reached differentiation. In all the experiments, mature adipocytes were used when 80% of cells appeared differentiated (8–10 total days). Unless otherwise specified, all other reagents were from Sigma-Aldrich (St Louis, MO, United States).

### Antiretroviral Drugs

Lopinavir (LPV) and ritonavir (RTV) were purchased from Santa Cruz Biotechnology (Dallas, TX, United States). These two PIs were dissolved in dimethyl sulfoxide (DMSO) and stored at -20°C, then diluted into culture media on the day of the experiment. Fully differentiated 3T3-L1 cells were treated with PIs for 48 h. LPV and RTV were used in combination at a 4:1 (LPV:RTV) ratio, as previously reported in studies on 3T3-L1 cells and human pre-adipocytes *in vitro* (Gallego-Escuredo et al., 2010; Zha et al., 2013), and consistent to the therapeutic regimen (Kumar et al., 2004). The final LPV:RTV doses were chosen on the basis of dose-response experiments (data not shown) and were 12 μM:3 μM and 16 μM:4 μM.

### Cells Viability Assays

Differentiated 3T3-L1 cells were seeded in 96-well culture plates and exposed to PIs for 48 h. At the end of treatment, cells were incubated with 3-(4,5-dimethylthiazol-2-yl)-2,5-diphenyl tetrazolium bromide (MTT, 5 mg/ml) at 37°C for 3 h. The formazan dye crystals were solubilized with 200 μl isopropanol. Absorbance was measured using a VICTOR3 spectrophotometer (Perkin Elmer Italia, Milano, Italy) at 570 nm wavelength. Experiments were repeated at least three times in separate days.

### Oil Red O Staining

3T3-L1 cells were plated in 24-well culture plates and exposed for 48 h to LPV:RTV combinations. At the end of the treatment, cells were washed twice with PBS and fixed with 10% formaldehyde in PBS for 90 min at 37°C. The fixative was removed, each well was washed twice with sterile water and stained with 200 μL of oil red O (ORO) solution for 2 h at 37°C. After the staining, cells were washed with sterile water and incubated with 200 μL of isopropanol; the absorbance was measured at 520 nm with a VICTOR3 spectrophotometer.

### Studies of Loss and Gain of Function: miRNA Inhibitor and miRNA Mimic Transfection Assays

MirVana^®^ miRNA inhibitor hsa-miR-218-5p, mirVana^®^ miRNA mimic hsa-miR-218-5p and mirVana^®^ Negative Control #1 were purchased from Ambion^®^ (Thermo Fisher Scientific, Waltham, MA, United States). Fully differentiated 3T3-L1 cells were treated with LPV:RTV and transfected with one of synthetic miRNAs using Lipofectamine RNAiMAX (Invitrogen, Carlsbad, CA, United States) according to the manufacturer’s protocol; 24 h after the transfection, culture medium was replaced with fresh culture medium. The effects of transfection of miRNA inhibitor or miRNA mimic or drug treatments, were evaluated after 48 h by ORO and real-time PCR. To ascertain the success of miRNA inhibitor transfection, 3T3-L1 cells were also transfected with mirVana^®^ miRNA inhibitor let-7 Positive Control (Ambion^®^). This miRNA inhibitor blocks miRNA let-7, an endogenous miRNA that usually down-regulates HMGA-2 gene expression. The levels of HMGA-2 mRNA were evaluated by real-time PCR.

### Real-Time PCR

Total RNA from fully differentiated and LPV:RTV treated 3T3-L1 cells was isolated with TRIzol (EuroClone, Pero, Italy) and quantified using a Nanodrop spectrophotometer (Thermo Fisher Scientific, Waltham, MA, United States). RT reaction was performed using either iScript^TM^ cDNA Synthesis Kit (Bio-Rad, Hercules, CA, United States) for lipin-1 and GLUT-4 mRNA expression (360 ng of total RNA), or TaqMan MicroRNA Reverse Transcription kit (Applied Biosystems, Thermo Fisher Scientific, Waltham, MA, United States) for miRNAs expression (10 ng of total RNA), according to the manufacturer’s instructions. Real-Time qPCR was performed using Taqman probes (Mm00550511_m1 for lipin-1, Mm00436615_m1 for GLUT-4, and assay 000521 for miRNA-218) and iTaq Universal Probe Supermix 2X (Bio-Rad, Hercules, CA, United States) in a 7900HT Fast Real-Time PCR System (Applied Biosystems, Thermo Fisher Scientific, Waltham, M, United States). The expressions of lipin-1 and GLUT-4 were normalized for β-actin, and that of miRNA-218 for U6 (Applied Biosystems, Thermo Fisher Scientific, Waltham, MA, United States), respectively.

### Statistical Analysis

Statistical analysis was performed using MATLAB (version R2010b; Mathworks). Analysis of variance (One Way ANOVA) was followed by Tukey-Kramer *t*-test. All results are expressed as mean ± standard error of the mean (SEM). A *p*-value of less than 0.05 was considered significant.

## Results

### Lopinavir:Ritonavir Treatment Does Not Affect 3T3-L1 Adipocytes Viability

Since PIs, including lopinavir and ritonavir, could affect adipose cell differentiation and replication, we used the MTT assay to ascertain whether different combinations of LPV:RTV could induce cell-toxicity and affect the viability of 3T3-L1 cells.

**FIGURE 1:**
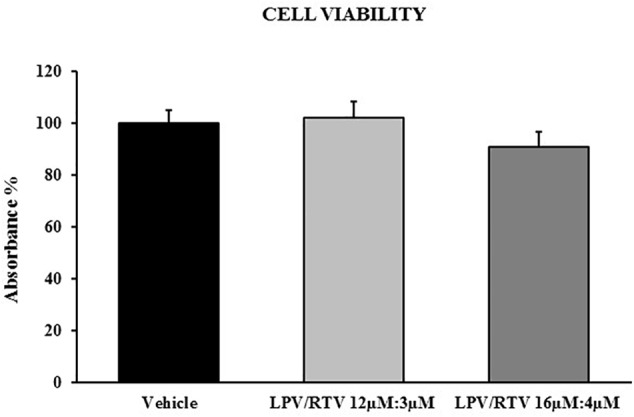
Effect of LPV:RTV treatment on viability of differentiated 3T3-L1 cells. Fully differentiated 3T3-L1 cells were treated for 48 h with LPV:RTV (12 μM:3 μM, 16 μM:4 μM) or with vehicle alone. Absorbance was measured at 570 nm. The values of absorbance were expressed relatively to absorbance measured in the vehicle group. Results are expressed as mean ± SEM and represent the mean of 18 determinations per group, obtained in three independent experiments.

Fully differentiated 3T3-L1 cells were exposed to two different combinations of LPV:RTV (12 μM:3 μM and 16 μM:4 μM); under both conditions, drug treatments did not significantly reduced cell viability compared to vehicle-treated cells (Figure 1).

### Lopinavir:Ritonavir Treatment Alters Lipid Accumulation in Mature Adipocytes

**FIGURE 2:**
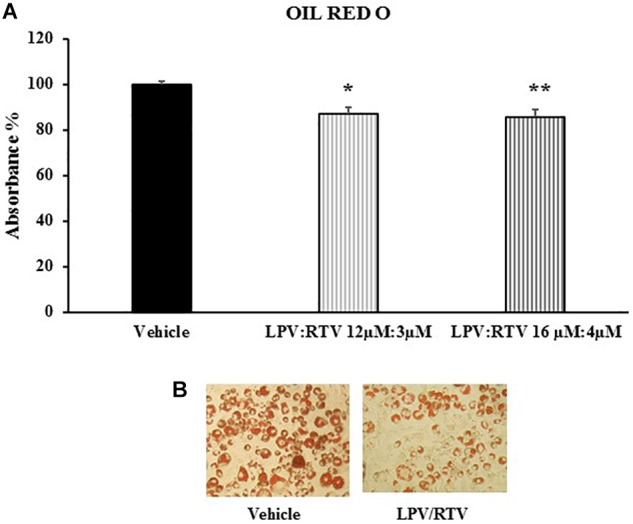
Effect of LPV:RTV treatment on lipid accumulation. Fully differentiated 3T3-L1 cells were treated as indicated in Figure 1. The intracellular lipids were stained with Oil Red O; the absorbance was measured at 520 nm. Absorbance was calculated as percent of that measured in vehicle group. Results are expressed as mean ± SEM and represent the mean of 18 determinations obtained in three independent experiments. ^∗^*p* < 0.05 and ^∗∗^*p* < 0.01 vs. vehicle **(A)**. Representative images for each treatment are shown **(B)**.

In order to verify whether mature 3T3-L1 adipocytes are sensitive to PIs treatment and could represent a useful *in vitro* model to reproduce the adipose tissue abnormalities caused by PIs regimens, the effects of LPV:RTV treatment on intracellular lipid accumulation were evaluated. ORO staining showed that exposure of mature adipocytes for 48 h to both LPV:RTV dose combinations caused a significant reduction of lipid accumulation compared to vehicle treated cells (-13.2% for 12 μM:3 μM and -14.5% for 16 μM:4 μM; *p* < 0.05 and *p* < 0.01 vs. vehicle, respectively) (Figure 2A,B).

### Effect of Lopinavir:Ritonavir Treatment on Lipin-1 mRNA Expression and miRNA-218 Expression in Mature 3T3-L1 Adipocytes

**FIGURE 3:**
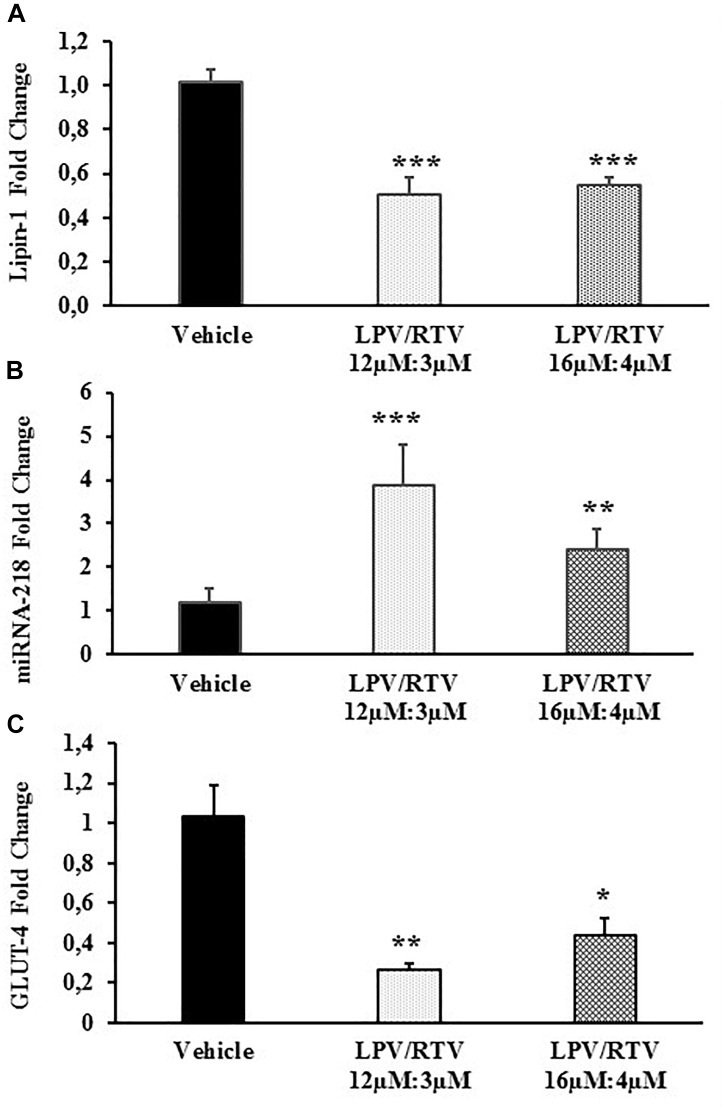
Effect of LPV:RTV treatment on lipin-1 and GLUT-4 mRNA expression and miRNA-218 levels. Fully differentiated 3T3-L1 cells were treated as indicated in the legend of Figure 1 levels of lipin-1 mRNA **(A)**, miRNA-218 **(B)** and GLUT-4 mRNA **(C)** were measured by real-time PCR. Levels of lipin-1 and GLUT-4 mRNA were normalized by those of β-actin; miRNA-218-5p levels were normalized using U6 levels. Values are the mean ± SEM of 18 determinations per group obtained in three independent experiments. ^∗^*p* < 0.05, ^∗∗^*p* < 0.01, and ^∗∗∗^*p* < 0.001 vs. vehicle.

Bioinformatic analysis showed that one of the predicted genes regulated by miRNA-218 is lipin-1. In order to define more precisely the inverse correlation between miRNA-218 and its putative target gene, lipin-1, mature 3T3-L1 adipocytes were treated with LPV:RTV (12 μM:3 μM, 16 μM:4 μM) for 48 h; lipin-1 mRNA levels and miRNA-218-5p expression were evaluated by real-time PCR. Lipin-1 mRNA levels were significantly lower after treatment with LPV:RTV compared to vehicle-treated cells (-51% for 12 μM:3 μM, and -47% for 16 μM:4 μM; *p* < 0.001 vs. vehicle). In contrast, miRNA-218-5p expression was higher in PIs treated cells than in the vehicle group (+220% for 12 μM:3 μM, and +81% for 16 μM:4 μM; respectively, *p* < 0.001 and *p* < 0.01 vs. vehicle) (Figure 3A,B), confirming the existence of an inverse relationship between lipin-1 and miRNA-218-5p expression in fully differentiated 3T3-L1 cells.

### Low Levels of Lipin-1 Correlate With Lower Expression of GLUT-4 mRNA in Mature 3T3-L1 Adipocytes

Since LPV:RTV treatment caused a reduction of lipin-1 mRNA levels, we attempted to ascertain whether it correlated to reduced levels of GLUT-4 mRNA in fully differentiated 3T3-L1. As expected, we found significantly reduced levels of GLUT-4 mRNA in LPV:RTV treated cells compared to vehicle treated cells (-74% for 12 μM:3 μM and -57% for 16 μM:4 μM; respectively, *p* < 0.01 and *p* < 0.05 vs. vehicle) (Figure 3C).

### miRNA-218 Targets Lipin-1 mRNA and Affects GLUT-4 Expression

The functional relevance of the specific miRNA/mRNA interaction was validated. In order to ascertain whether miRNA-218 could regulate lipin-1 mRNA, fully differentiated 3T3-L1 cells treated with LPV/RTV 12 μM:3 μM for 48 h were transfected with either specific inhibitors or mimics for miRNA-218-5p.

**FIGURE 4:**
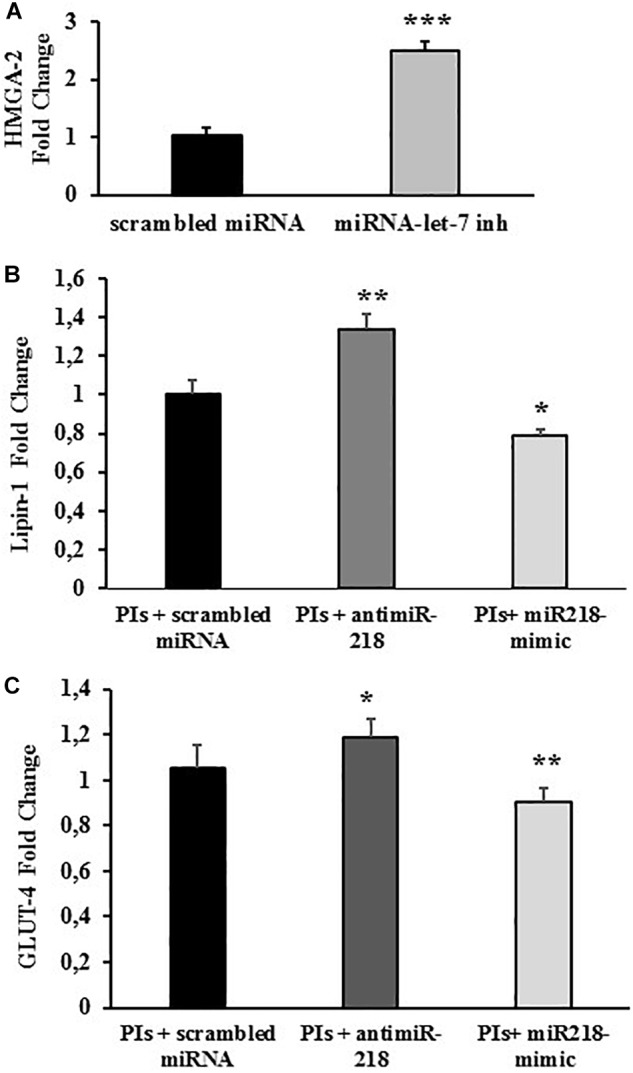
miRNA-218 levels affect lipin-1 and GLUT-4 mRNA expression. Fully differentiated 3T3-L1 cells treated with LPV:RTV (12 μM:3 μM) (PIs) for 48 h or with vehicle alone, were transfected with: **(A)** scrambled miRNA or miRNA-let-7 inhibitor; **(B,C)** miRNA-218 inhibitor or miRNA-218 mimic or with scrambled miRNA. mRNA levels of HMGA-2 **(A)** lipin-1 **(B)** and GLUT-4 **(C)** were determined by real-time PCR 48 h after the transfection. Values are the mean ± SEM of at least 18 determination obtained in three independent experiments. ^∗^*p* < 0.05, ^∗∗^*p* < 0.01 and ^∗∗∗^*p* < 0.001 vs. control (PIs+ scrambled miRNA).

The efficiency of the miRNA inhibitor transfection was evaluated by co-transfecting the cells with miRNA-let-7 inhibitor Positive Control, an endogenous miRNA that down-regulates HMGA-2 gene expression. HMGA-2 is a ubiquitously expressed non-histone chromatin protein that modulates gene expression through changes in chromatin architecture. As expected, transfection with miRNA-let-7 inhibitor significantly increased the expression of HMGA-2 (+150% vs. negative control; *p* < 0.01), attesting the efficacy of the adopted procedure (Figure 4A).

Lipin-1 mRNA levels were significantly higher in cells treated with PIs and miRNA-218 inhibitor compared to cells treated with PIs and a scramble control miRNA (+ 34% vs. scrambled control; *p* < 0.01). Conversely, mature adipocytes exposed to LPV:RTV and transfected with a miRNA mimic of miRNA-218 expressed lower levels of lipin-1 mRNA compared to cells transfected with a scrambled miRNA (-22% for miRNA-218 mimic vs. scrambled control; *p* < 0.05) (Figure 4B). These experiments clearly suggested a functional inverse relationship between miRNA-218 and its putative target gene lipin-1.

GLUT-4 mRNA expression changed after treatment with 12 μM:3 μM PIs and concurrent transfection with either miRNA 218-inhibitor or miRNA 218-mimic showing the same trends observed for lipin-1. In fact, the miRNA-218 inhibitor induced a significant increase of GLUT-4 mRNA levels (+17% vs. scrambled; *p* < 0.05) whereas the miRNA-218 mimic lowered them significantly (-12% vs. scrambled; *p* < 0.01) compared to controls (Figure 4C).

### Effects of Loss and Gain of Function of miRNA-218 on Lipid Accumulation

The biological relevance of the loss of function of miRNA-218 in 3T3-L1 mature adipocytes was studied by measuring the effects of the transfection of a miRNA-218 inhibitor and a miRNA-218 mimic on lipid accumulation.

**FIGURE 5:**
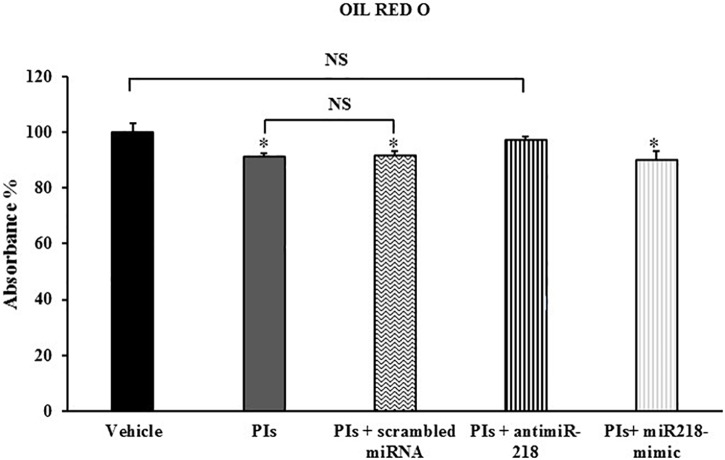
Effects of loss and gain of function of miRNA-218 on lipid accumulation. Fully differentiated 3T3-L1 cells were treated with vehicle, or with PIs alone or in combination with scrambled miRNA or miRNA-218 inhibitor or miRNA-218 mimic. The intracellular lipids were stained with Oil Red O; the absorbance was measured at 520 nm and expressed as percent of control group. Results are the mean ± SEM of at least 18 determinations obtained in three independent experiments. ^∗^*p* < 0.05 vs. vehicle.

As expected, treatment with LPV:RTV 12 μM:3 μM caused a significant decrease of lipid accumulation compared to control cells (-9% PIs vs. vehicle; *p* < 0.05). Transfection with scrambled miRNA did not affect lipid accumulation. Transfection with the miRNA-218 mimic reduced the capability of 3T3-L1 cells to store lipid droplets (-10% PIs+ miRNA mimic vs. vehicle; *p* < 0.05), without causing significant additive effects compared to PIs alone. Conversely, inhibition of miRNA-218 activity by the specific miRNA inhibitor reversed PIs effects and improved significantly the ability of the cells to store lipid droplets (Figure 5).

## Discussion

Metabolic alterations and changes in the distribution of adipose tissue are typical features of HIV-associated metabolic syndrome, including LS, a serious problem occurring in patients taking anti-HIV drugs. Since the pathophysiology of LS due to cART is an irreversible process, even after drug withdrawal, elucidation of the underlying mechanisms inducing LS is a matter of particular importance (Finkelstein et al., 2015). LS was first recognized in patients on PIs treatment (Carr and Cooper, 1998). PIs have revolutionized HIV treatment paradigms and dramatically increased the life expectancy of the HIV-positive population, although their administration is associated with several adverse effects, including dyslipidaemia, insulin resistance and hyperglycaemia (Holstein et al., 2001; Calza et al., 2004; Calvo and Martinez, 2014). Among PI class, lopinavir boosted with ritonavir, is frequently associated with metabolic disorders (Calvo and Martinez, 2014). Recent guidelines for initial therapy recommend use of integrase strand transfer inhibitors (INSTIs) regimens in order to avoid the negative effects on metabolism. However, PIs are currently part of cART especially in patients harboring multi drug-resistant viruses (Huhn et al., 2017) and exposure to ritonavir-boosted PIs is widely represented in patients with a long history of treatment (Murnane et al., 2017).

Several studies have demonstrated alteration on adipocyte differentiation and lipid content induced by PIs (Caron-Debarle et al., 2010). It is known that *in vitro* LPV and RTV activate endoplasmic reticulum (ER) stress in adipocytes through proteasome inhibition, induce mitochondrial dysfunction and ROS production, alter cell differentiation and lipid content, and disrupt the expression of key regulatory genes involved in lipid metabolism (Parker et al., 2005; Capel et al., 2012; Zha et al., 2013). Indeed, they interfere with intracellular processes regulating glucose uptake in major insulin-responsive tissues. LPV/RTV strongly inhibited glucose uptake by altering GLUT-4 activity in adipocytes (Giralt et al., 2006; Noor et al., 2006). However, their precise effects in LS has been only partially defined.

Altered miRNA processing and Dicer expression have been proposed to have a role also in LS (Mori et al., 2014). In this regards, exposure of the adipocytes to the PI saquinavir resulted in upregulation of miRNAs, some of them have been related to obesity, insulin resistance and lipolysis (Polus et al., 2017); nevertheless, the functional significance of these miRNAs is not defined.

To the best of our knowledge, this is the first research to demonstrate (i) a direct link between the miRNA regulation process in adipocytes and LPV/RTV treatment and (ii) a functional relationship between a specific miRNA, the miRNA-218 and its molecular target, the lipin-1 gene. Indeed, in differentiated 3T3–L1 cells PIs treatment reduced lipin-1 mRNA expression and augmented miRNA-218 expression, decreasing their capability to store lipid droplets. The “loss and gain of function” studies allowed to demonstrate that miRNA-218 targets the lipin-1 gene, thereby providing new knowledge in the regulation of lipin-1 mRNA expression by a specific miRNA.

The interactions between a specific miRNA and its mRNA target are difficult to assess. In this setting, “loss-of-function” studies (in which a specific endogenous mature miRNA function can be inhibited using antisense oligoribonucleotides), combined with “gain of function” studies (in which a specific endogenous mature miRNA function can be increased using an exogenous miRNA mimic) constitute a validated and consolidated approach (Kuhn et al., 2008). Beside the methodological procedure, it is relevant the choice of the cell line. 3T3-L1 adipocytes and SGBS pre-adipocytes are widespread used *in vitro* models for providing detailed insights into the molecular mechanisms governing adipogenesis (Capel et al., 2012; Zha et al., 2013; Ahonen et al., 2019; Cave and Crowther, 2019). Although 3T3-L1 adipocytes have some limitations and could not be completely representatives of *in vivo* conditions, they possess some advantages: are homogenous in terms of cellular population and their cells are all at the same differentiation stage, giving a homogeneous response to treatments. For these reasons, 3T3-L1 have been widely utilized for *in vitro* studies of anti-HIV drugs effects (Minami et al., 2011; Manente et al., 2012; Parker et al., 2005) on growth and differentiation and to investigate the role of different miRNAs in adipogenic processes (Lin et al., 2009; Lee et al., 2011; Ahn et al., 2013; Yun et al., 2015).

In differentiated 3T3-L1 cells exposed to different PIs doses, the inhibition of miRNA-218 restored mRNA levels of lipin-1 and allowed the recovery of adipogenic functionality. By contrast, transfection of 3T3-L1 with the miRNA-218-5p mimic had opposite effect, demonstrating that miRNA-218 negatively regulates lipin-1 mRNA expression. Analog approach has been utilized in other study where the knockdown of miR-34a prevented the senescence of vascular endothelial cells induced by LPV/RTV exposure (Zhan et al., 2016). These results, consistent with those obtained in subcutaneous adipose tissue of lipodystrophic HIV patients on anti-HIV drugs (Squillace et al., 2014), namely miRNA-218-5p over-expression and lipin-1 mRNA down-regulation, corroborates the use of 3T3-L1 adipocytes treated with PIs as a good *in vitro* model to investigate some open aspects of lipodystrophy cART-induced.

To date, there is no report attesting a PIs direct effect on miRNA-218 and lipin-1 mRNA expression. Lipin-1 has been identified as a gene required for normal adipose tissue development. It plays an essential role in adipocyte differentiation and function (Peterfy et al., 2005; Sembongi et al., 2013), and promotes C/EBPα and PPARγ_2_ gene expression, which in turn controls the function of other genes expressed in adipose tissue, as well as the fatty acids binding protein 4 or phosphoenolpyruvate carboxykinase (Chen et al., 2015). In addition to this role during early adipogenesis, lipin-1 is also required for correct lipid droplet biogenesis (Sembongi et al., 2013). Mutations of the lipin-1 gene leads to LS in the mouse (Peterfy et al., 2001) and adipocyte differentiation is completely blocked by siRNA-mediated knock-down of lipin-1 (Chen et al., 2015). Both lipin-1 expression levels in human adipose tissue and lipin-1 genetic polymorphisms are associated with alterations in body mass index and insulin sensitivity in different human cohorts (Donkor et al., 2008; Wiedmann et al., 2008; Chang et al., 2010). In contrast to the effects of lipin-1 deficiency, selective overexpression of lipin-1 mRNA levels in adipocytes induced obesity in transgenic mice, with increased size and triglyceride content in adipocytes (Csaki and Reue, 2010). Interestingly, the obese adipose-specific lipin-1 transgenic mice were characterized by improved glucose and insulin homeostasis, suggesting that increased capacity for triglyceride synthesis within adipocytes via lipin-1 is metabolically favorable (Csaki and Reue, 2010), perhaps by partitioning fatty acids into adipose tissue and away from ectopic storage in other tissues (Zhang and Reue, 2017).

HIV patients on cART regimens who are affected by metabolic syndrome and lipodystrophy have decreased lipin-1 mRNA expression in subcutaneous adipose tissue, compared to HIV-infected patients without metabolic complications and lipodystrophy. In addition, an inverse correlation between lipin-1 mRNA expression and miRNA-218 levels was observed (Squillace et al., 2014). Nevertheless, until now the mechanisms underlying this relationship has not been investigated.

The understanding of miRNA-218 role remains elusive; it has been proposed as a tumor suppressor gene, and among its targets have been recognized genes related to apoptosis, cellular migration, tumor invasion and cellular proliferation (Yamamoto et al., 2013; Yamasaki et al., 2013; Cao et al., 2015; Dong et al., 2015; Han et al., 2015; Zhang et al., 2015).

A recent paper showed a relationship between miRNA-218 and the function of GLUT (glucose transporter)-1 across tumoral bladder cells: the overexpression of miRNA-218 could have contributed to decreased sensitivity of bladder cancer cells to cisplatin by inhibiting GLUT-1 expression, a key rate-limiting factor in the transport of glucose in these cancer cells (Li et al., 2017). A primary feature of insulin resistance in LS (Cortes and Fernandez-Galilea, 2015) is reduced GLUT-4 insulin-mediated glucose uptake in muscle and adipose tissue (Gallego-Escuredo et al., 2013). In muscle, this effect is mainly due to impaired recruitment of GLUT-4 to the plasma membrane despite normal GLUT-4 expression (Shepherd and Kahn, 1999); in adipocytes, however, this effect seems to be associated with depressed expression of GLUT-4 (Govers, 2014). Reduced levels of lipin-1 in mature adipocytes are linked to decreased expression of GLUT-4 mRNA (Chen et al., 2015), the insulin-stimulated glucose transporter specific for the adipocytes (Govers, 2014). Given the relationship between miRNA-218 and lipin-1, we speculated that miRNA-218 could also affect GLUT-4 gene expression. Accordingly, we report for the first time that LPV/RTV treatment reduces GLUT-4 mRNA expression in differentiated 3T3-L1 cells and that, interestingly, this effect is linked once again to miRNA-218 expression, as confirmed by loss-of function studies. Consistent with our results, also saquinavir influences gene expression of the GLUT-4 glucose transporter in adipocytes, inducing its down-regulation (Polus et al., 2017).

In view of these outcomes, we hypothesize that the metabolic alterations of adipose tissue observed in HIV patients could be ascribed to PIs effects on lipin-1 mRNA expression; this effect is most likely indirectly mediated by an increase of miRNA-218 levels, as revealed by miRNA functional studies. These increased miRNA-218 levels, indeed, negatively regulate lipin-1 mRNA expression, causing alterations of his functionality. Increased miRNA-218 levels could be also responsible for a reduced mRNA expression of GLUT-4, suggesting that miRNA-218 could be involved in the mechanisms by which cART induces metabolic syndrome and insulin resistance in HIV patients.

In conclusion, the results of this study could help the comprehension of mechanisms underlying the LS cART-induced and open new perspectives of investigation.

## Author Contributions

EB wrote the manuscript and put the idea. EB, CS, LR, LM, and RM conducted the study. EB, AT, NS, and VL helped to design the study. EB, CS, and AT analyzed the data. RO, AG, GB, AT, NS, and VL revised the manuscript.

## Conflict of Interest Statement

The authors declare that the research was conducted in the absence of any commercial or financial relationships that could be construed as a potential conflict of interest.
